# Oxidant-Mediated Protein Amino Acid Conversion

**DOI:** 10.3390/antiox8020050

**Published:** 2019-02-25

**Authors:** Yuichiro J. Suzuki

**Affiliations:** Department of Pharmacology and Physiology, Georgetown University Medical Center, Washington, DC 20007, USA; ys82@georgetown.edu; Tel.: +1-202-687-8090; Fax: +1-202-687-8825

**Keywords:** amino acid, carbonylation, oxidation, protein, reactive oxygen species, redox

## Abstract

Biological oxidation plays important roles in the pathogenesis of various diseases and aging. Carbonylation is one mode of protein oxidation. It has been reported that amino acids that are susceptible to carbonylation are arginine (Arg), proline (Pro), lysine, and threonine residues. The carbonylation product of both Arg and Pro residues is glutamyl semialdehyde. While chemically the oxidation reactions of neither Pro to glutamyl semialdehyde nor Arg to glutamyl semialdehyde are reversible, experimental results from our laboratory suggest that the biological system may drive the reduction of glutamyl semialdehyde to Pro in the protein structure. Further, glutamyl semialdehyde can be oxidized to become glutamic acid (Glu). Therefore, I hypothesize that biological oxidation post-translationally converts Arg to Pro, Arg to Glu, and Pro to Glu within the protein structure. Our mass spectrometry experiments provided evidence that, in human cells, 5–10% of peroxiredoxin 6 protein molecules have Pro-45 replaced by Glu. This concept of protein amino acid conversion challenges the dogma that amino acid sequences are strictly defined by nucleic acid sequences. I propose that, in the biological system, amino acid replacements can occur post-translationally through redox regulation, and protein molecules with non-DNA coding sequences confer functions.

## 1. The Rationale for the Concept of Oxidant-Mediated Protein Amino Acid Conversions

**Figure 1:**
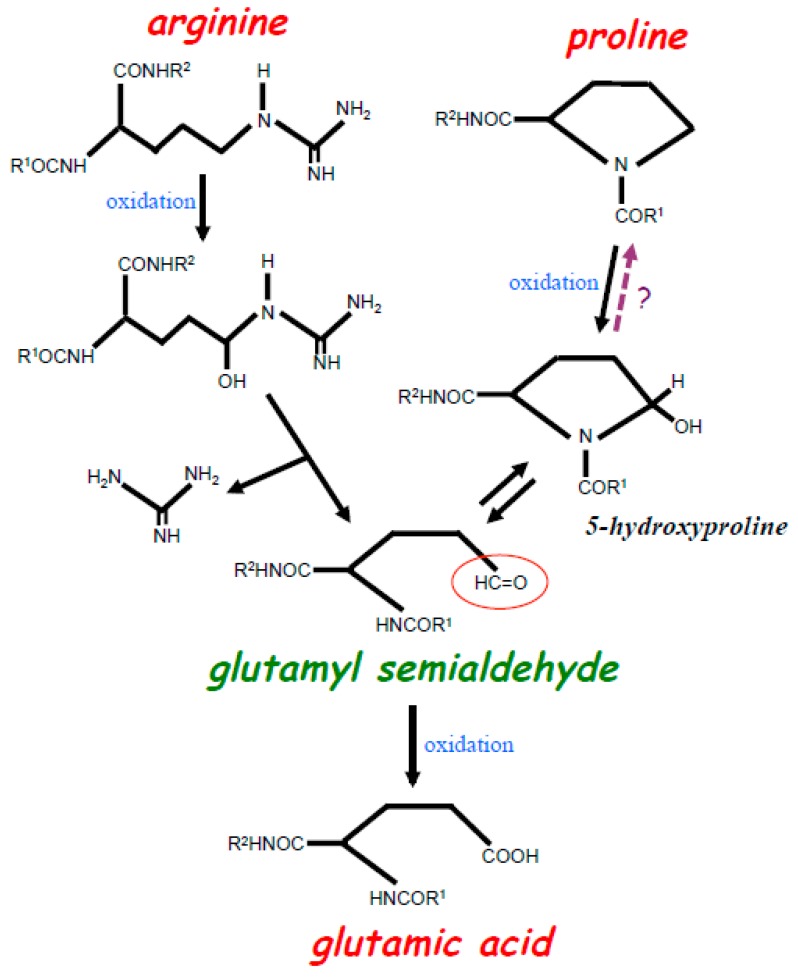
Scheme depicting the oxidation of Arg and Pro residues in the protein structure. Iron-catalyzed oxidation of both Arg and Pro residues results in the formation of glutamyl semialdehyde with a carbonyl group (red circle). Oxidation of Arg results in the loss of a bulky amino group-containing structure during the formation of glutamyl semialdehyde. Oxidation of Pro first forms 5-hydroxyproline, which is further oxidized into glutamyl semialdehyde. Studies of purified proteins indicate that the oxidation of Pro to 5-hydroxyproline is irreversible, yet our experiments with live cells suggest that this reaction may be reversible (as indicated with?). Glutamyl semialdehyde can further be oxidized into Glu.

Biological oxidation and oxidative stress promoted by reactive oxygen species (ROS) play important roles in the pathogenesis and progression of many diseases, as well as in the aging process [1]. ROS can oxidize various biological molecules including proteins, DNA, lipids, and small molecules [1]. Protein carbonylation is one mode of protein oxidation, in which structures of amino acid side chains get modified, resulting in the introduction of new carbonyl groups [2,3]. It has been reported that the amino acids that are susceptible to carbonylation are arginine (Arg), proline (Pro), lysine, and threonine residues [4,5]. Oxidation of both Arg and Pro produces glutamyl semialdehyde that contains a carbonyl group, which can further be oxidized into glutamic acid (Glu) (Figure 1). Figure 1 also depicts that chemically, the oxidation reactions of neither Pro to glutamyl semialdehyde nor Arg to glutamyl semialdehyde are reversible [4].

Our laboratory previously discovered that protein carbonylation plays a role in ligand/receptor-mediated cell signaling [6,7,8]. We also noted that the ligand-mediated protein carbonylation exhibited transient kinetics in cultured cells; the promotion of carbonylation occurring at about 10 min and returning to the baseline by 30 min. We named this process “protein decarbonylation” [6]. We further showed that the addition of thiol reductants to cell lysates or tissue homogenates resulted in decreased protein carbonyl content [6,9]. However, the addition of thiol reductants to purified proteins did not decrease the protein carbonyl content. These results indicate that protein carbonyls are not reduced in the absence of other cellular components and that cells contain catalysts for the reduction of protein carbonyls. We also showed that the inactivation of cellular enzymes by heating or knocking down a cellular reductant, glutaredoxin 1 (Grx1), inhibited protein decarbonylation [9]. 

We hypothesized that protein decarbonylation may involve the reduction of glutamyl semialdehyde back to Pro in the protein structure (Figure 1). This event could occur simply through the two-step electron reduction of glutamyl semialdehyde to 5-hydroxyproline then to Pro, although Amici et al. [4] described that 5-hydroxyproline is not usually reduced to Pro through their studies of purified proteins and peptides. We attributed that the catalysis by biological factors, perhaps Grx1, may allow for the reduction of 5-hydroxyproline to Pro. In contrast, it is not likely that glutamyl semialdehyde can go back to Arg because a large amino group-containing moiety is lost. Since both Arg and Pro become glutamyl semialdehyde in the process of protein carbonylation and possibly biological factors catalyze Pro carbonylation to be reversible, I argue that Arg residues can be converted into Pro through oxido-reduction reactions in the biological system, leading to my hypothesis for the concept of the protein amino acid conversion. Further, since the oxidation of glutamyl semialdehyde should produce Glu, Arg-to-Glu and Pro-to-Glu conversions could also occur in the protein structures. Thus, through glutamyl semialdehyde, Arg, Pro, and Glu could interchangeably occur in a given amino acid residue in a process similar to protein engineering that manipulates the DNA sequences, but in this case post-translationally. 

## 2. Experimental Evidence for the Occurrence of Protein Amino Acid Conversions in the Biological System

**Figure 2:**
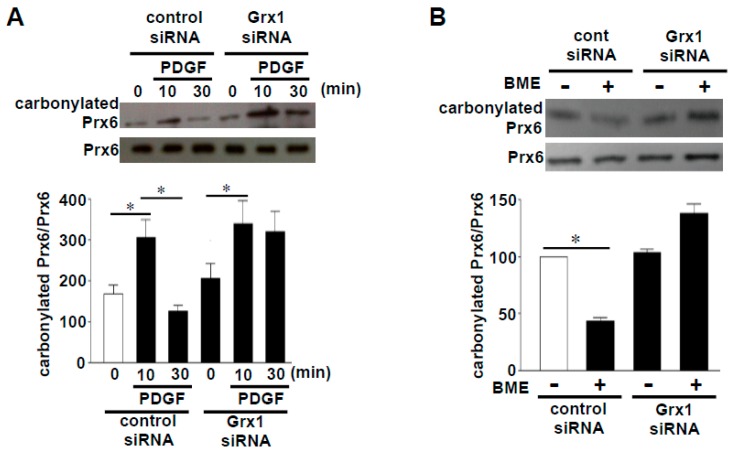
Effects of siRNA knockdown of glutaredoxin 1 (Grx1) on peroxiredoxin 6 (Prx6) decarbonylation. (**A**) Cultured human smooth muscle cells were transfected with Grx1 siRNA, followed by stimulation with platelet-derived growth factor (PDGF). Cell lysates were prepared, derivatized with 2,4-dinitrophenylhydrazine (DNPH), immunoprecipitated with the DNPH antibody, and immunoblotted with the Prx6 antibody [9]. (**B**) Cultured human smooth muscle cells were transfected with Grx1 siRNA, and cell lysates were prepared. Cell lysates were then treated with or without 2% (w/v) BME, derivatized with DNPH, immunoprecipitated with the DNPH antibody, and immunoblotted with the Prx6 antibody [9]. Bar graphs represent means ± SEM of the ratio of carbonylated Prx6 to Prx6 protein expression. The symbol * denotes values significantly different from each other at *p* < 0.05.

Through the proteomic approach, we identified some proteins that undergo protein decarbonylation [9]. These proteins included peroxiredoxin 6 (Prx6), an antioxidant enzyme that eliminates peroxides by utilizing the catalytic cysteine at position 47 [10,11]. As shown in Figure 2A, the treatment of cultured human smooth muscle cells with platelet-derived growth factor, which has been shown to elicit oxidant signaling [12,13], caused an increase in carbonylated Prx6 at 10 min, and the decarbonylation occurred within 30 min. Treating cell lysates with a thiol reductant, BME, also caused the decarbonylation of Prx6 (Figure 2B), suggesting that decarbonylation is thiol reduction-dependent [9]. siRNA knockdown of a cellular thiol reductant, Grx1, indeed inhibited the Prx6 decarbonylation in both systems (Figure 2A,B) [9].

**Figure 3:**
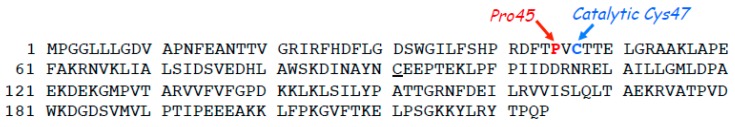
Amino acid sequence of human Prx6 protein molecule. Proline 45 (Pro45) and catalytic cysteine 47 (Cys47) are indicated by arrows.

Because of our interest in protein decarbonylation that was found to be electron reduction-dependent [6,9], we initially attempted to identify the occurrence of the Arg-to-Pro conversion using mass spectrometry. We enriched Prx6 by immunoprecipitation from cultured human smooth muscle cells. Prx6 immunoprecipitation samples were then processed for tryptic digestion and analyzed by mass spectrometry [14]. While we have not yet detected Arg-to-Pro conversions consistently occurring in our samples, we reproducibly obtained data that support the Pro conversion to Glu in Prx6 [14]. This Pro-to-Glu conversion occurs at Pro 45 residue in the human Prx6 protein molecule, which is within the conserved catalytic sequence encompassing cysteine 47 (Figure 3). In living cells, 5–10% of Prx6 protein molecules were found to have this Pro45-to-Glu conversion [14]. This conversion appears to be regulated post-translationally, but not due to DNA mutation, because the treatment of cells with hydrogen peroxide (H_2_O_2_) for only 10 min increased the Pro45-to-Glu conversion to occur in 70% of Prx6 molecules [14].

These experimental results have provided the evidence that Pro45 within the Prx6 protein molecule is converted to Glu post-translationally, strengthening the concept that the oxidant-mediated amino acid conversions occur at functionally important amino acid residues of protein molecules. 

## 3. Role of Oxidant-Mediated Protein Amino Acid Conversion in Biology

### 3.1. Challenging the Dogma of DNA Strictly Defining the Primary Structures of Proteins

The central dogma of molecular biology describes the concept that the sequence information is transferred from DNA to RNA, then to protein [15]. Therefore, it is generally stated that the order of amino acids in the protein is determined by DNA. DNA mutation, either naturally or artificially, could result in amino acid replacement in the protein structure. Further, cells are capable of editing RNA to produce protein sequences that are not defined by DNA [16]. However, to my knowledge, there have not been any biological mechanisms that describe changing the amino acid sequences of already synthesized proteins. 

While a variety of post-translational modifications of proteins have been described, they are all processes that modify amino acids to structures that are distinct from fundamental amino acids. As molecular biology techniques have advanced, all the protein sequences are now deduced from DNA sequences, and it is assumed that DNA-coding sequences are the primary structure of proteins. Therefore, it is intriguing to think that amino acids can be converted to different amino acids post-translationally. It is also exciting that biological redox processes regulate such processes, providing another role of redox reactions in the biological system and another form of redox regulation. 

**Figure 4:**
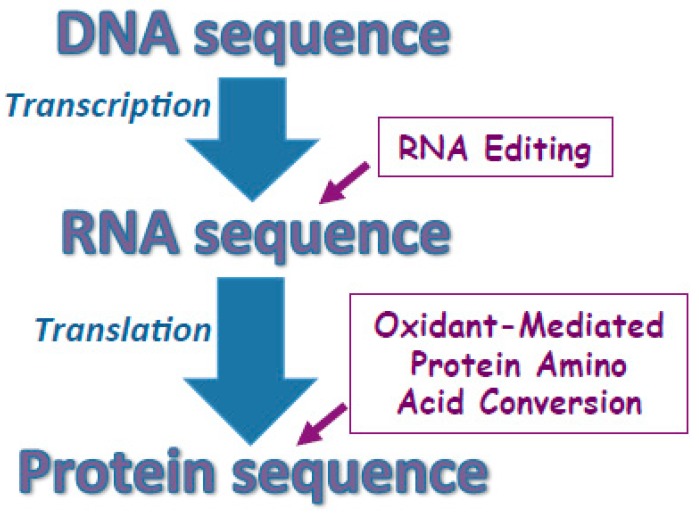
Proposed mechanisms for defining protein amino acid sequences. Protein amino acid sequences are largely defined by the sequence of DNA that is transcribed to mRNA then translated to the protein sequence. In some cases, RNA editing changes the sequence of mRNA thereby altering the protein amino acid sequence not to encode the DNA sequence completely. I proposed that the amino acid sequences can also be altered post-translationally through the oxidant-mediated protein amino acid conversions.

While the concept of the described oxidant-mediated amino acid conversion is limited to Arg-to-Pro, Arg-to-Glu and Pro-to-Glu conversions, the implications of these small changes could be enormous. Since Pro plays a crucial role in determining the tertiary structures of proteins, either the introduction or the deletion of Pro through amino acid conversions should have tremendous influence on protein structures and functions. The Arg-to-Glu conversion also changes the charge of the amino acid side chain that would be likely to have a significant impact. Figure 4 describes a proposed, revised view of how amino acid sequences may be defined by considering the possibility of post-translationally-regulated oxidant-mediated protein amino acid conversions.

### 3.2. Challenging the Dogma of Proteostasis of Oxidized Proteins

**Figure 5:**
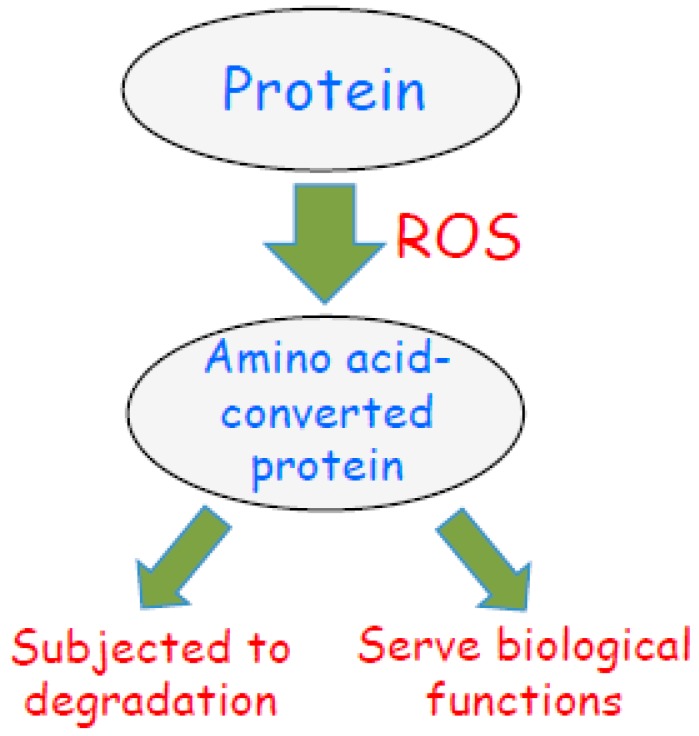
Scheme depicting the fate of amino acid-converted proteins in response to biological oxidation. Reactive oxygen species (ROS) are produced during oxidative stress or oxidant signaling. ROS promote amino acid conversions as described in this article. Some of these amino acid-converted proteins are subjected to protein degradation as a mechanism of oxidative stress. I propose that amino acid-converted proteins also possess biologic functions as nature intended.

The concept of oxidant-mediated protein amino acid conversion may also promote the thinking that oxidized proteins may not be merely degraded in the biological system, as is often thought. It is generally believed that the role of protein carbonylation is to mark the oxidized proteins as damaged molecules so that such proteins can be degraded [17,18,19]. However, we should also consider the possibility that the oxidized proteins, particularly those whose amino acids have been converted, may be functional proteins with alternative amino acid sequences, tertiary structures, and functions that have been designed by nature (Figure 5).

## 4. Limitations and Future Directions

While the concept of oxidant-mediated amino acid conversion provides an exciting new mechanism of redox regulation that may challenge the dogma of biology, further work is needed to support this hypothesis. While our experiments provided initial evidence that a functionally important Pro residue in the Prx6 protein molecule may be converted to Glu, these mass spectrometry results are based on the +31.990 Da mass shift that could be due to some other post-translation modifications [14].

If my hypothesis for the occurrence of oxidant-mediated protein amino acid conversions turns out to be incorrect and amino acids encoded by the nucleotide sequences strictly define the primary protein structures, then it would indicate that the biological system possesses a remarkable mechanism to prevent the occurrence of protein structures that defies the DNA sequence.

If amino acid conversions within the protein structure indeed occur, then we need to question how such processes are regulated. The biological system does contain the means to convert free amino acids including the Pro-to-Glu conversion via glutamyl semialdehyde, and these mechanisms are catalyzed by certain enzymes [20,21]. Further work is needed to determine whether these catalysts are also capable of affecting amino acids within the protein structure.

## 5. Conclusions

In summary, I herein propose an exciting concept that a given amino acid can be replaced by another amino acid through redox-regulated post-translational modifications.
